# Control of Magnetic Shape Anisotropy by Nanopillar Dimensionality in Vertically Aligned Nanocomposites

**DOI:** 10.1021/acsaelm.4c00371

**Published:** 2024-04-18

**Authors:** Marijn W. van de Putte, Dmytro Polishchuk, Nicolas Gauquelin, Johan Verbeeck, Gertjan Koster, Mark Huijben

**Affiliations:** † MESA+ Institute for Nanotechnology, University of Twente, P.O. Box 217, 7500 AE Enschede, Netherlands; ‡ Institute of Magnetism of the National Academy of Sciences of Ukraine and Ministry of Education and Science of Ukraine, 03142 Kyiv, Ukraine; § Electron Microscopy for Materials Science (EMAT), Uweniversity of Antwerp, 2020 Antwerp, Belgium

**Keywords:** magnetic anisotropy, vertically aligned nanocomposite, nanopillar, epitaxy, LSMO, ZnO

## Abstract

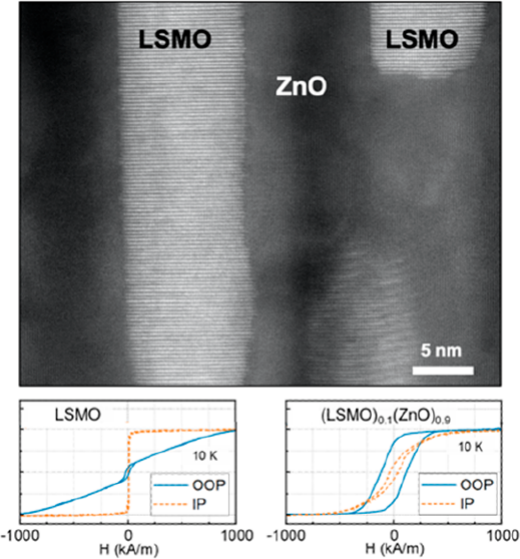

Perpendicular magnetic anisotropy forms the foundation of the current data storage technology. However, there is an ever-increasing demand for higher density data storage, faster read-write access times, and lower power consuming storage devices, which requires new materials to reduce the switching current, improve bit-to-bit distributions, and improve reliability of writing with scalability below 10 nm. Here, vertically aligned nanocomposites (VANs) composed of self-assembled ferromagnetic La_0.7_Sr_0.3_MnO_3_ (LSMO) nanopillars in a surrounding ZnO matrix are investigated for controllable magnetic anisotropy. Confinement of LSMO into nanopillar dimensions down to 15 nm in such VAN films aligns the magnetic easy axis along the out-of-plane (i.e., perpendicular) direction, in strong contrast to the typical in-plane easy axis for strained, phase pure LSMO thin films. The dominant contribution to the magnetic anisotropy in these (LSMO)_0.1_(ZnO)_0.9_ VAN films comes from the shape of the nanopillars, while the epitaxial strain at the vertical LSMO:ZnO interfaces exhibits a negligible effect. These VAN films with their large, out-of-plane remnant magnetization of 2.6 μB/Mn and bit density of 0.77 Tbits/inch^2^ offer an interesting strategy for enhanced data storage applications.

## 1 Introduction

The continuous expansion of global interconnectivity (IoT, 5G communication, remote monitoring, etc.) goes hand in hand with tremendous advancements in data storage. There is an ever-increasing demand for higher density data storage, faster read-write access times, and lower power consuming storage devices. In magnetic storage technology, binary digital data are stored in the magnetization directions of tiny regions on a magnetic thin film. Initially, longitudinal recording was used for magnetic recording in which the magnetic bits lie in the plane of the thin film medium. About two decades ago perpendicular recording was introduced where the magnetic bits are perpendicular to the plane of the recording media due to perpendicular magnetic anisotropy (PMA).^1,2^ The dramatic increase in storage density by more than 1 order of magnitude was accompanied by an enhanced stability of the data storage domains due to the weaker in-plane demagnetizing field.

Nonvolatile spin-transfer torque (STT) magnetoresistive random access memory (MRAM) is one of the most promising data storage methods by using a spin-polarized current to change the magnetization direction.^3,4^ A common commercial STT-MRAM structure uses an in-plane magnetic tunnel junction (MTJ), which means that the magnetization of the magnetic layers lies in the plane of the material. However, STT-MRAM devices with a more optimized MTJ structure have been proposed where the magnetic moments are perpendicular to the thin film magnetic material, i.e., termed perpendicular MTJ. Perpendicular STT-MRAM requires a much lower switching current, which decreases further on decreasing the cell size. Furthermore, it also facilitates simpler designs, reduces manufacturing cost, offers excellent scalability and, therefore, has the potential to become a leading storage technology with scalability below 10 nm. Current focus is on the development of new PMA materials to reduce the switching current, improve bit-to-bit distributions, and improve reliability of writing. This requires developing PMA materials with low magnetic damping, high spin-polarization (for high magnetoresistance), and high magnetic exchange stiffness, which are typically interrelated.

La_0.7_Sr_0.3_MnO_3_ (LSMO) is a pseudocubic perovskite oxide material (lattice parameter *a* = 3.889 Å^5^) exhibiting a large magnetic moment (3.7 μ_B_/Mn)^6^ and a high Curie temperature (∼370 K)^6,7^ and is, therefore, a very interesting candidate for room temperature, and above, applications such as sensors,^8^ catalysts,^9^ biomedical treatments,^10^ microelectronics,^11^ and data storage.^12^ Enhancement of electrochemical, magnetoresistive, and ferromagnetic properties has been previously explored by controlling the dimensionality of LSMO in a variety of material architectures such as 0D core–shell nanoparticles,^13^ 1D nanowires,^14^ 2D planar films,^15^ and 3D vertically aligned nanocomposites.^16^

Although magnetocrystalline anisotropy is in the case of LSMO thin films negligibly small,^17^ the strain state of an LSMO thin film has a large effect on its magnetic response. An in-plane tensile strained LSMO phase (e.g., grown on substrates with larger cubic crystal structure, for example on SrTiO_3_ (STO) (*a* = 3.905 Å)) will exhibit an in-plane magnetic easy axis,^18,19^ whereas an in-plane compressively strained LSMO phase (e.g., grown on substrates with smaller cubic crystal structure, such as LaAlO_3_ (LAO) (*a* = 3.82 Å)) will exhibit a magnetic easy axis in the out-of-plane direction.^20^ Furthermore, shape anisotropy influences the magnetic behavior such that the magnetic easy axis tends to align perpendicular to the reduced dimension of the shape, e.g., in the plane of a 2D ultrathin film or along the axis of a 1D nanowire. However, it has been shown that in LSMO nanowires with a large aspect ratio, a tensile strain perpendicular to the nanowire axis causes the easy axis to lie perpendicular to the nanowire axis, overcoming the shape anisotropy.^14^ Detailed investigation of magnetic shape anisotropy has been previously performed in thin films of magnetic pillar structures fabricated through a top-down approach by etching channels into an Al_2_O_3_ matrix and depositing Co into the pillar-shaped holes.^21^ These films of Co pillars showed a magnetic response dominated by the shape anisotropy, where the easy axis aligns with the pillar direction for nanopillars with an aspect ratio of more than 1.6. However, magnetic shape anisotropy has not been explored for pillared LSMO structures to determine the influence of nanopillar dimensionality on magnetic behavior.

Here, vertically aligned nanocomposites composed of LSMO nanopillars in a surrounding ZnO matrix are investigated for controllable magnetic anisotropy. Self-assembled vertically aligned nanocomposite (VAN) thin films formed by two immiscible oxides can exhibit specific properties not available in single-phase materials.^16,22,23^ The immiscibility of the two phases forms the foundation of the self-assembly procedure, resulting in highly ordered nanopillar/matrix structures. VANs composed of LSMO and ZnO have previously been studied for their low-field magnetoresistance (LFMR)^24−28^ in which ZnO nanopillars are introduced in the LSMO film matrix. In contrast, in this work, LSMO nanopillars are formed in the ZnO film matrix for which the magnetic behavior is affected by the shape effect and the strain at the vertical LSMO:ZnO interfaces. It is shown that confinement of LSMO from planar films into nanopillar dimensions down to 15 nm switches the magnetic easy axis from in-plane to out-of-plane (i.e., perpendicular) orientation. The dominant contribution to the magnetic anisotropy in these (LSMO)_0.1_(ZnO)_0.9_ VAN films comes from the shape of the nanopillars, while the epitaxial strain at the vertical LSMO:ZnO interfaces exhibits a negligible effect.

## 2 Experimental Section

Thin films of pure LSMO, pure ZnO, and VAN ratios, (LSMO)_0.3_(ZnO)_0.7_ and (LSMO)_0.1_(ZnO)_0.9_, were grown by pulsed laser deposition on (100)-oriented SrTiO_3_ (STO) substrates using stoichiometric targets. The growth temperature was kept at 850 °C with an oxygen pressure of 0.27 mbar. A laser frequency of 1 Hz was used for the pure LSMO and ZnO thin films to ensure high quality,^15^ while 5 Hz was used for the (LSMO)_0.3_(ZnO)_0.7_ and (LSMO)_0.1_(ZnO)_0.9_ thin films to achieve the self-assembled VAN formation.^25^ The crystal structure, surface morphology, composition, and thin film thickness were investigated by X-ray diffraction (XRD, PANalytical X’Pert PRO), atomic force microscopy (AFM, Bruker ICON Dimension Microscope), and scanning electron microscopy (SEM, Zeiss Merlin). More detailed structural and compositional analysis was performed through high-resolution scanning transmission electron microscopy (HR-STEM) and energy dispersive X-ray spectroscopy (EDX) measured on a Titan 80–300 aberration-corrected electron microscope operated at 300 kV. Electrical and magnetic analysis was carried out using a Dynacool PPMS system (Quantum Design) equipped with vibrating sample magnetometry (VSM) in the temperature range 5–380 K. To determine the Curie temperature (*T*_C_), the thin films were field-cooled to 10 K under an 800 kA/m (1 T) magnetic field, followed by a careful reduction of the field to 8 kA/m, after which the films were heated at 3 °C/min while constantly measuring the magnetization. In-plane and out-of-plane *M*–*H* hysteresis loops were collected by mounting the sample parallel or perpendicular to the applied magnetic field. The thin films were field cooled to 10 K under a 1600 kA/m (2 T) magnetic field after which the *M*–*H* loops were measured by sweeping the field between −1600 and +1600 kA/m at selected temperatures. For the analysis of the magnetization, values for the film thicknesses were used as extracted from SEM images, including an estimated error of 5%.

## 3 Results and Discussion

### 3.1 Structural Properties

**Figure 1:**
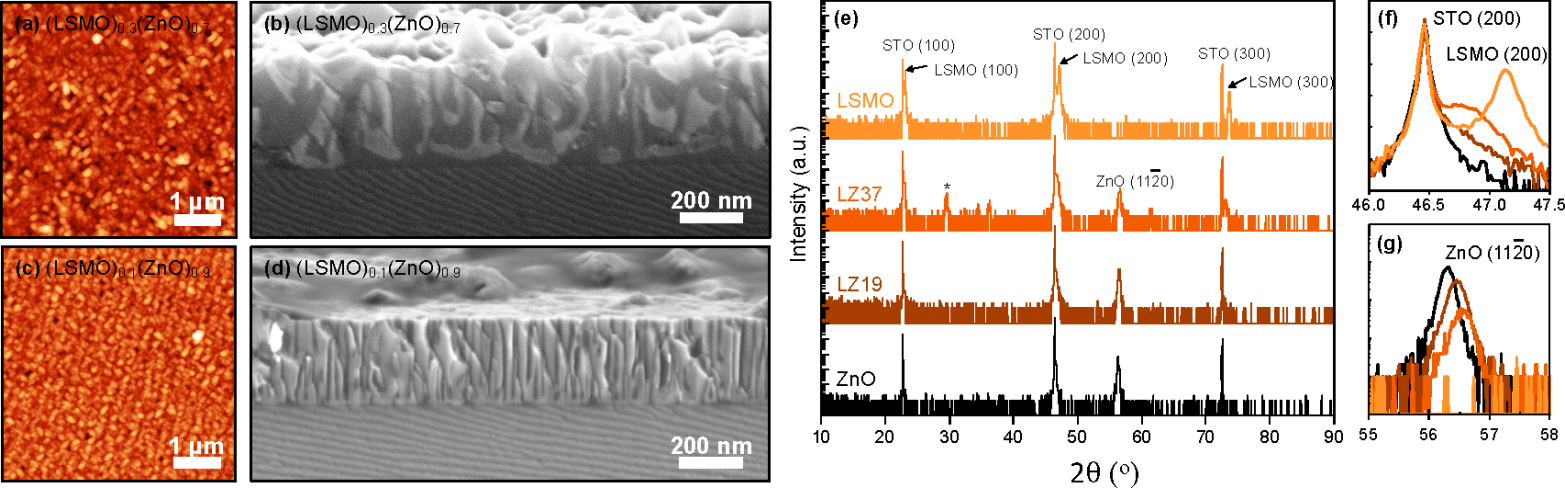
AFM and SEM images of (a, b) (LSMO)_0.3_(ZnO)_0.7_ (LZ37) and (c, d) (LSMO)_0.1_(ZnO)_0.9_ (LZ19) VAN thin films. (e) Symmetric θ–2θ XRD measurements of VAN thin films, as well as LSMO and ZnO single films, on STO (100) substrates. Symbol (*) indicates small La_2_O_3_ impurity phase. (f, g) Respectively, the LSMO (200) peak and ZnO (11–20) peak in detail.

The nanocomposite formation through self-assembly was studied in detail for two different LSMO:ZnO VAN ratios (3:7 and 1:9) and compared to those of single LSMO and ZnO layers. To evaluate the surface morphology, AFM analysis was performed, which indicates significant differences (Figure 1a and c). The VAN film with a large LSMO contribution (3:7), similar to previous studies, exhibits square-like features on the surface. The surface features are about 50–100 nm in lateral size and seem to align diagonally, which has been shown previously to match the (0001) plane of the ZnO phase.^27^ The RMS roughness is about 30 nm, which is significantly more than only a few nanometers for the single LSMO and ZnO layers (not shown). However, the VAN film with a low LSMO contribution (1:9) exhibits smaller surface features of about 20–50 nm in lateral size and seems to align with the step edges of the step-and-terrace surface of the underlying STO substrate. The RMS roughness is also significantly reduced to about 10 nm, much closer to surfaces of single LSMO and ZnO layers. The surface morphologies are confirmed by cross-sectional SEM analysis, as shown in Figure 1b and d. It can be clearly observed that the lighter LSMO regions are much narrower and exhibit a more vertical alignment in the 1:9 ratio VAN film, most likely induced by the enhanced strain of the surrounding, darker ZnO regions.

X-ray diffraction analysis demonstrates the realization of single oriented LSMO and ZnO phases within the VAN thin films, similar to single LSMO or ZnO films on (100)-oriented STO substrates (Figure 1e). The cubic LSMO structure aligns epitaxially with the cubic structure of the STO substrate LSMO (100) || STO (100), while the ZnO hexagonal wurtzite structure also aligns epitaxially with the cubic STO substrate ZnO (11–20) || STO (100). The (LSMO)_0.3_(ZnO)_0.7_ VAN thin film exhibits a small impurity phase, most likely La_2_O_3_, which originates from the starting target composition.

A fully epitaxially, in-plane strained LSMO film on a (100) STO substrate exhibits a reduced *c*-axis of 3.853 Å (Figure 1f), in good agreement with the literature.^15^ When the LSMO phase is incorporated in VAN thin films by the surrounding ZnO structure, the LSMO structure relaxes close to its bulk value of 3.889 Å. The *c*-axis of LSMO becomes 3.881 and 3.891 Å for VAN thin films of respectively (LSMO)_0.3_(ZnO)_0.7_ and (LSMO)_0.1_(ZnO)_0.9_. The LSMO (200) peak in Figure 1f shifts toward the STO (200) peak and exhibits a dramatic reduction in intensity due to the limited 10% contribution in the VAN thin film. The corresponding ZnO (11–20) peak exhibits a strong increase in intensity, and the VAN thin film containing 90% ZnO contribution is close to the full ZnO thin film (Figure 1g). The shift in ZnO (11–20) peak position toward higher θ angles for reduced ZnO contribution in VAN thin films indicates a reduced distance between (11–20) planes. The ZnO phase changes from a fully relaxed, pure film on a STO substrate to an out-of-plane compressed phase in the nanocomposite, driven by the differences in lattice matching at the interfaces between the ZnO and LSMO phases. With a lattice matching of 5:6 for ZnO:LSMO as previously observed^16^ at the vertical interfaces between LSMO and ZnO, a lattice mismatch of 0.46% is still present, which is compensated by tensile straining the LSMO and compressing the ZnO phases in the out-of-plane direction.

**Figure 2:**
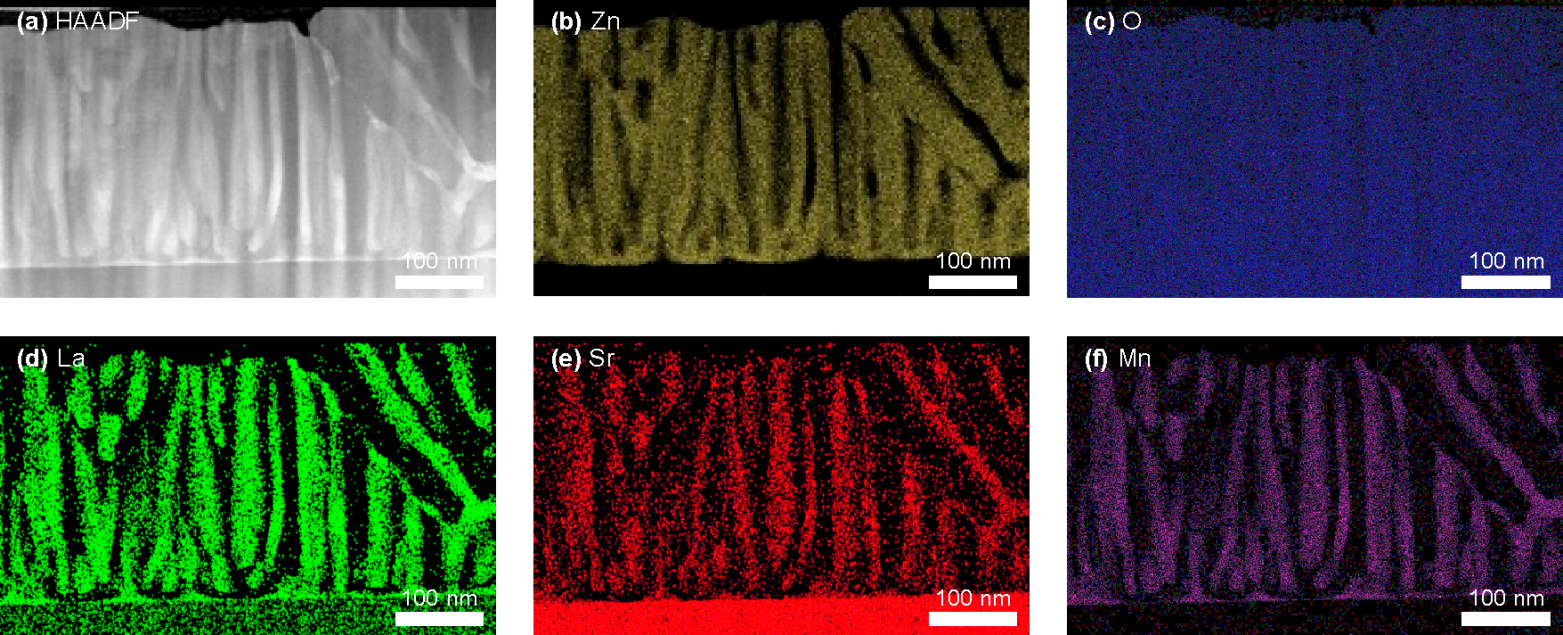
STEM-EDX cross-sectional maps showing the different elements within the (LSMO)_0.1_(ZnO)_0.9_ VAN thin film with (a) HAADF overview and (b) zinc, (c) oxygen, (d) lanthanum, (e) strontium, and (f) manganese contributions.

Detailed analysis of the structural ordering within the nanocomposite thin films was studied by scanning transmission electron microscopy. High-angle annular dark-field (HAADF) imaging shows the vertical alignment within the nanopillar-matrix structures; see Figure 2a. From EDX spectroscopy, it can be seen clearly that there is a separation of the elements of the LSMO and ZnO phases in the nanocomposite film, with the La, Sr, and Mn elements occurring in the same pillars, while the Zn mostly occurs in the other regions (Figure 2b–f). The thickness of the STEM lamella is about 10 nm, which results in overlap regions where both LSMO and ZnO phases seem to be present. The width of the LSMO pillars in this (LSMO)_0.1_(ZnO)_0.9_ VAN thin film is estimated to be between 10 and 30 nm.

**Figure 3:**
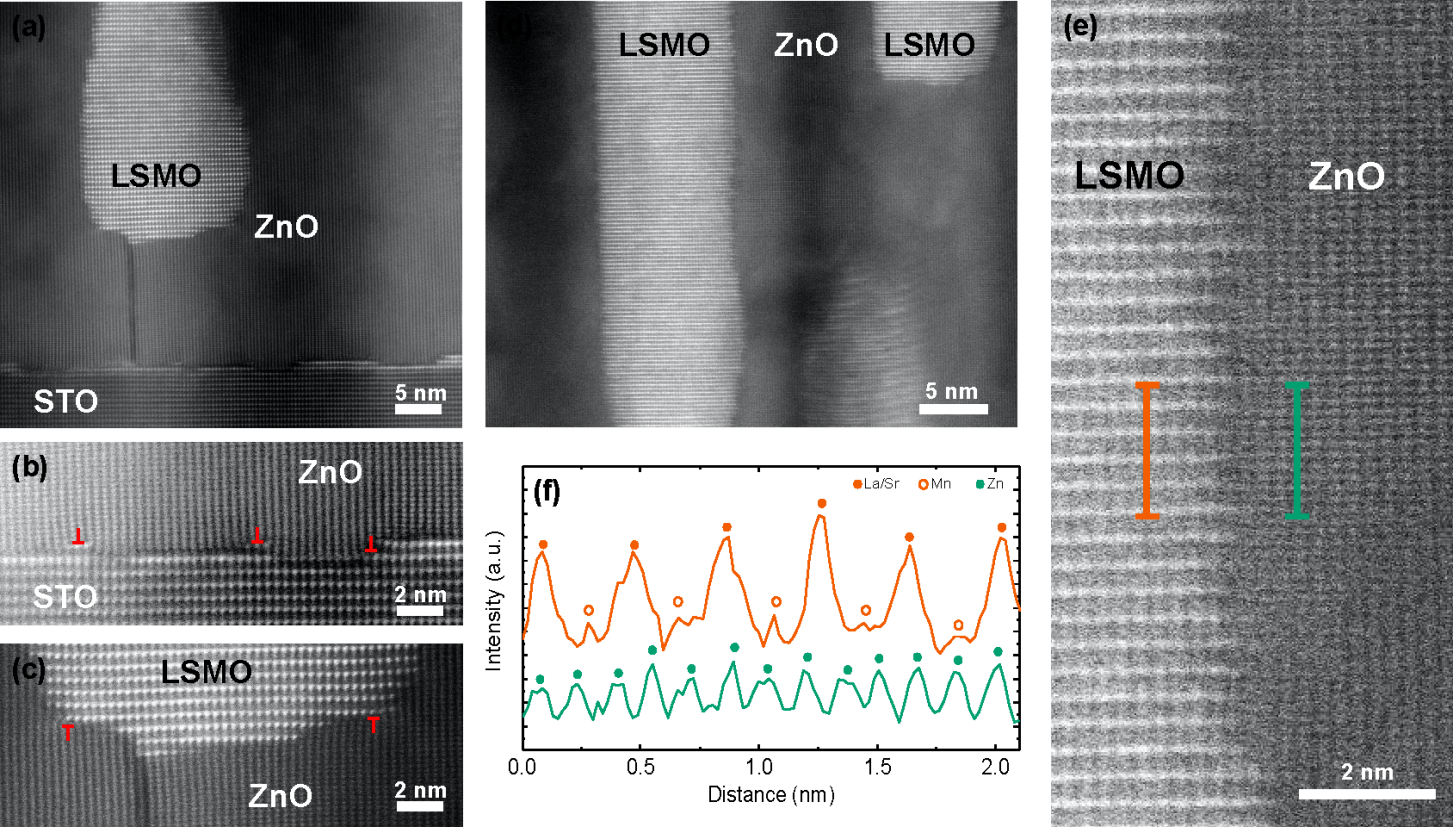
STEM cross-sectional images of a (LSMO)_0.1_(ZnO)_0.9_ VAN thin film displaying (a) the LSMO pillar in the ZnO matrix, (b) the interface between the ZnO matrix and the underlying STO substrate, and (c) the horizontal and (d) vertical interfaces between LSMO and ZnO regions. Edge dislocations are marked with a red T. (e) Detailed analysis of the alignment of the LSMO and ZnO structures at the vertical interface, for which (f) intensity profiles of the LSMO and ZnO phases are shown along the lines in part e.

High-resolution STEM analysis along the [110] zone axis of the STO substrate demonstrates the epitaxial alignment of the LSMO and ZnO phases to each other, as well as to the underlying STO substrate; see Figure 3a. It can be seen that at the interface to the STO substrate, regions exist where the ZnO phase connects directly, while in other regions an intermediate, interfacial LSMO layer (bright contrast) exists just one to three atomic layers thin (Figure 3a). A dark vertical line can be seen in the ZnO phase (Figure 3a), which is most likely a grain boundary or a line defect in the crystalline phase. Above this vertical line, an LSMO nanopillar has formed, most likely nucleating on the defect in the ZnO phase (Figure 3c). A closer look at the horizontal interface to the substrate shows that the lattice mismatch between the ZnO and STO crystal structures causes stress in the ZnO phase, which is relieved by the formation of edge dislocations, which are marked in Figure 3b. The theoretical mismatch between ZnO and STO is as high as 5.8% along the *c*-axis of ZnO, which should correspond to a defect formed once every 100/5.8 = 17 unit cells, which matches well with distances of 14 and 20 unit cells between defects observed in Figure 3b. Similar defects are also observed for the horizontal interface between the ZnO matrix and the LSMO nanopillar in Figure 3c. Interestingly, the defects seem to occur in the ZnO phase, although it is the LSMO phase that was grown on top.

At the vertical interfaces between the LSMO nanopillars and the surrounding ZnO matrix the corresponding crystal structures align themselves epitaxially as well; see Figure 3d. In Figure 3e it can be seen that the vertical interfaces are not completely sharp; however, a clear lattice matching of the two crystal structures can be observed in good agreement with a previous study.^16^ The intensity line profile of LSMO exhibits strong peaks corresponding to the La and Sr atoms, while in between small peaks can be observed corresponding to the Mn atoms (Figure 3f). The intensity line profile of ZnO shows one type of peak corresponding to the Zn atoms. The six La peaks mark the five unit cells of LSMO, which match to six unit cells of ZnO as marked by the 13 Zn peaks. This lattice matching of 5:6 unit cells of LSMO:ZnO is observed previously^16^ and minimizes the total strain at these vertical interfaces.

### 3.2 Electrical and Magnetic Properties

**Figure 4:**
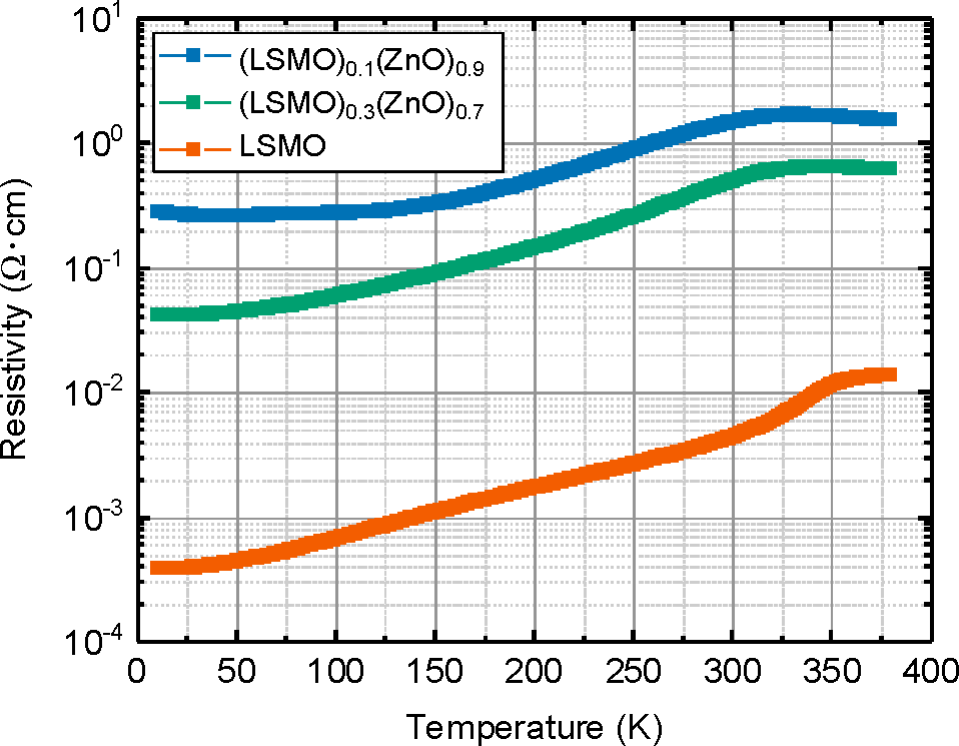
Temperature-dependent in-plane electrical transport behavior for a phase pure LSMO film as well as (LSMO)_0.3_(ZnO)_0.7_ and (LSMO)_0.1_(ZnO)_0.9_ VAN films.

The in-plane electrical transport behavior has been investigated in detail for the VAN films as well as the phase pure LSMO and ZnO thin films for the temperature range of 5–380 K; see Figure 4. The pure LSMO film exhibits clear metallic behavior, in good agreement with a previous study,^15^ while the pure ZnO film shows insulating behavior over the full temperature range with resistivities outside the capability of the used measurement system. Interestingly, the VAN films also exhibit clear metallic behavior, although resistivities are about 1–2 orders of magnitude higher compared to phase pure LSMO films. It seems logical that the (LSMO)_0.1_(ZnO)_0.9_ VAN film with an LSMO contribution of only 10% displays the highest resistivity. However, the observed metallic behavior indicates that a percolation path still exists for the mobile charge carriers between the individual LSMO nanopillars through the intermediate ZnO matrix.

**Figure 5:**
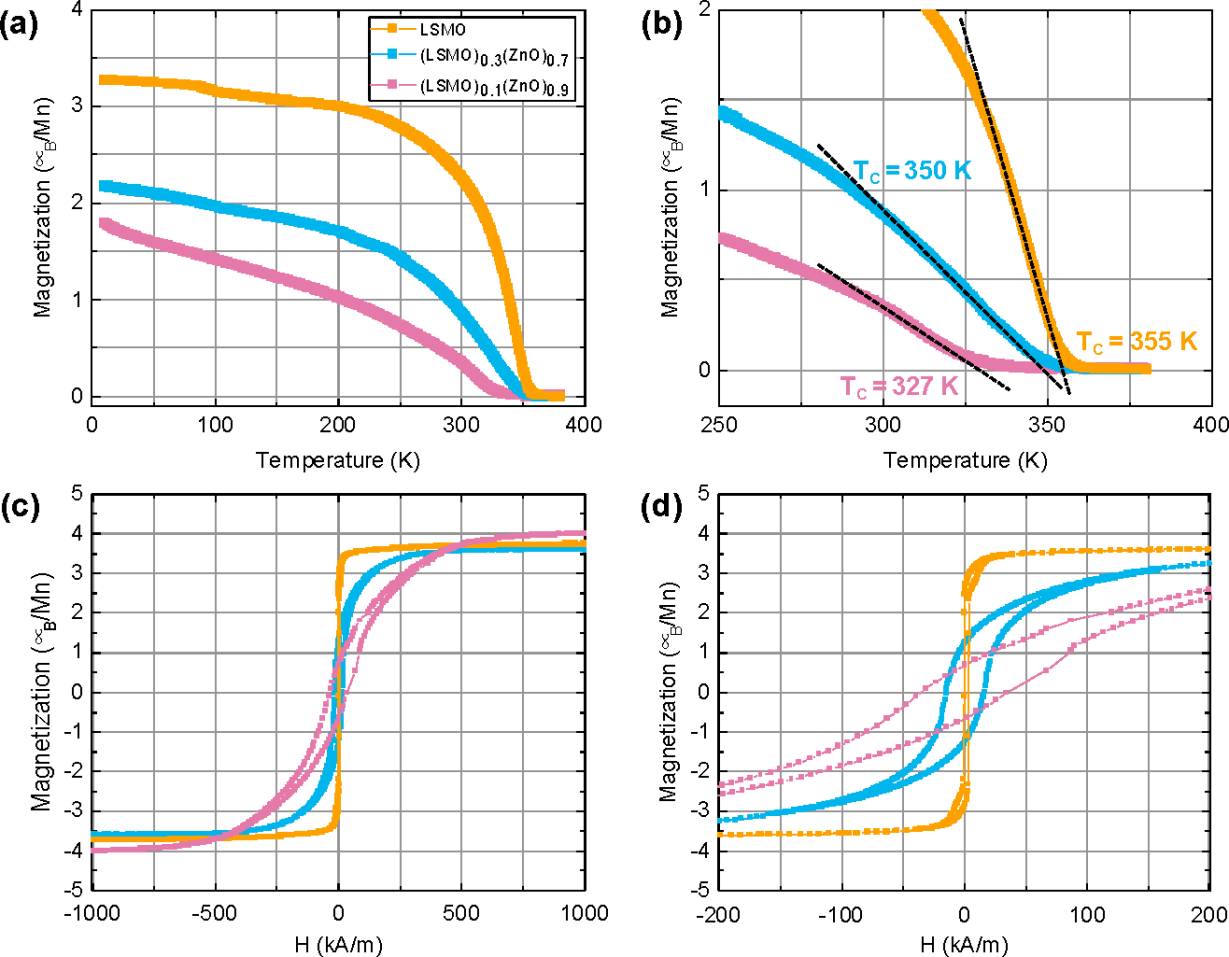
Temperature-dependent magnetization measurements for a phase pure LSMO film as well as (LSMO)_0.3_(ZnO)_0.7_ and (LSMO)_0.1_(nO)_0.9_ VAN films. *M* vs *T* in the in-plane direction at a magnetic field of 8 kA/m (a) over the full temperature range and (b) a zoom-in around the Curie temperatures. (c) *M* vs *H* hysteresis loops in the in-plane direction at 10 K with (d) a zoom-in around zero applied field.

The magnetic behavior of a tensile-strained LSMO film on a (100)-oriented STO substrate is a well-known and studied system.^15,19,20^ The in-plane (IP) tensile strain forces the easy axis in-plane, while the shape anisotropy strengthens this by also forcing the easy axis to lie in the film plane. All magnetic domains switch upon applying a small, in-plane oriented magnetic field, resulting in a small coercive field (*H*_c_) and a saturation magnetization (*M*_sat_) close to the theoretical magnetization of 3.7 μB/Mn for bulk LSMO. The temperature-dependent magnetic behavior of the phase pure LSMO film exhibits a Curie temperature (*T*_c_) of about 355 K (Figure 5), in good agreement with a previous study.^15^ For the VAN films with LSMO contributions of 30% or 10% the *T*_c_ is reduced to 350 and 327 K, respectively. This can be explained by the loss of long-range structural ordering of the LSMO phase in the VAN films due to the presence of vertical interfaces with ZnO, resulting in a weaker double-exchange interaction.^16^ However, the Curie temperatures of these VAN films are higher than those of related VAN films in previous studies^24,25,29^ with even higher LSMO contributions. Those previous studies with a 50:50 LSMO:ZnO ratio resulted in VAN films with a specific checkerboard formation and Curie temperature of 303 K,^29^ while their pure LSMO films also exhibited a Curie temperature of 353 K, in very close agreement with our results for pure LSMO films. The reduction of the Curie temperature was previously related to the decrease in the LSMO volume fraction. However, reduction of LSMO volume cannot be the origin, as it was demonstrated previously that high Curie temperatures can be achieved in pure LSMO films down to layer thicknesses of only 5 nm.^15^

The in-plane magnetic hysteresis (M vs H) loops show saturation magnetization of about 3.7 ± 0.2 μ_B_/Mn for the phase pure LSMO film, in good agreement with previous study.^15^ The (LSMO)_0.3_(ZnO)_0.7_ VAN film with an LSMO contribution of 30% results in a similar 3.6 ± 0.2 μ_B_/Mn, while the (LSMO)_0.3_(ZnO)_0.7_ VAN film with an LSMO contribution of only 10% leads to 4.1 ± 0.2 μ_B_/Mn, even higher than the theoretical maximum of 3.7 μ_B_/Mn. The magnetic contribution of impurities or secondary phases can be excluded by detailed XRD and STEM analysis. However, similar high magnetization values have been previously reported for studies on LSMO single crystals, polycrystals, and epitaxial thin films.^6,30,31^ The presence of a large number of vertical interfaces with locally strained LSMO regions in these VAN films could play an important role.

The phase pure LSMO film displays a very small coercive field *H*_c_ of 0.9 kA/m, as seen typically for in-plane tensile-strained LSMO thin films.^15^ However, the (LSMO)_0.3_(ZnO)_0.7_ and (LSMO)_0.1_(ZnO)_0.9_ VAN films exhibited significantly larger *H*_c_ values of respectively 15.4 and 35.7 kA/m (Table 1). Such increase of the coercive field is related to the reduced dimensions of the LSMO phase, as observed also for LSMO thin films with decreasing thickness.^15^ A similar effect has also been observed in cobalt nanopillar arrays where a smaller diameter of the pillars resulted in a larger coercive field.^21^ In the LSMO:ZnO VAN films, this could be caused by the pinning of magnetic domains at the vertical LSMO:ZnO interfaces due to local structural distortions. This would result in a larger energy requirement for switching of the single magnetic domain in the nanopillar^32^ and, therefore, leads to an increased coercive field. As the density of the vertical LSMO:ZnO interfaces increases for VAN films with a larger ZnO contribution, an expected increase of the domain pinning for (LSMO)_0.1_(ZnO)_0.9_ VAN films is in good agreement with experimental observations.

**Table 1:** Summary of the Magnetic Parameters of the Phase Pure LSMO and VAN Films at 10 K

	LSMO		(LSMO)_0.3_(ZnO)_0.7_		(LSMO)_0.1_(ZnO)_0.9_	
	IP	OOP	IP	OOP	IP	OOP
*H*_c_ (kA/m)	0.9 ± 0.2	10.5 ± 0.1	15.4 ± 1.4	23.2 ± 0.2	35.7 ± 0.6	116 ± 4
*H*_an_ (kA/m)	0.6 ± 0.1	1106 ± 10	27 ± 5	310 ± 8	207 ± 8	167 ± 8
*M*_**r**_ (μ_B_/Mn)	2.8 ± 0.2	0.42 ± 0.03	1.2 ± 0.1	0.29 ± 0.02	0.66 ± 0.05	2.6 ± 0.2
*M*_sat_ (μ_B_/Mn)	3.7 ± 0.2		3.6 ± 0.2		4.1 ± 0.2	
*T*_C_ (K)	355 ± 1		350 ± 1		327 ± 1	

**Figure 6:**
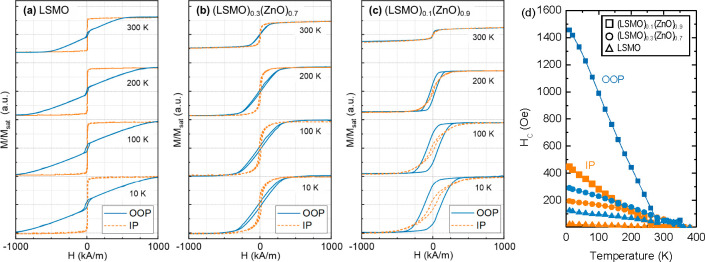
*M*–*H* hystersis loops at specific temperatures for in-plane (IP) and out-of-plane (OOP) orientations of (a) a phase pure LSMO film, (b) an (LSMO)_0.3_(ZnO)_0.7_ VAN film, and (c) an (LSMO)_0.1_(ZnO)_0.9_ VAN film. (d) Temperature dependence of the coercive field (*H*_c_) for all films.

Detailed analysis is performed for the magnetic response of the thin films in the in-plane (IP) and out-of-plane (OOP) directions at specific temperatures; see Figure 6. The tensile-strained, phase pure LSMO film shows a clear easy axis in the IP direction and a hard axis in the OOP direction, as expected for tensile-strained LSMO films.^19,20^ The magnetic response of the (LSMO)_0.3_(ZnO)_0.7_ VAN film shows a clear distinction between IP and OOP magnetization, where the easy axis is still aligned in the IP direction. The hysteresis loop is more rounded as compared to the phase pure LSMO film, indicating that most magnetic domains switch at low applied fields, but larger fields are required to fully magnetize the VAN film. The OOP direction has a much steeper slope than for the pure LSMO film, indicating this VAN film exhibits a reduced anisotropy. This is a direct result of the nanopillar-matrix architecture of the VAN film, where many ZnO regions have been introduced in the LSMO thin film, breaking the shape anisotropy. In the (LSMO)_0.1_(ZnO)_0.9_ VAN film the ZnO contribution is further increased, and the alignment of the magnetic domains (i.e., nanopillars) along the OOP direction becomes easier. The direction of the easy axis for the (LSMO)_0.1_(ZnO)_0.9_ VAN film is not directly evident from the qualitative comparison of the IP and the OOP loops, although the OOP direction seems to be most likely. Interestingly, the remnant magnetization and coercive field in the OOP direction (Table 1) become respectively 2.6 μB/Mn and 116 kA/m for the (LSMO)_0.1_(ZnO)_0.9_ VAN film, which is significantly higher than the phase pure LSMO film (0.42 μB/Mn and 10.5 kA/m) and (LSMO)_0.3_(ZnO)_0.7_ VAN film (0.29 μB/Mn and 23.2 kA/m). Such magnetic behavior of the LSMO nanopillars is required for robust data storage applications and limits susceptibility to random field fluctuations.

To fortify any claims about the easy axes, the anisotropy field (*H*_an_) is determined by fitting a linear relation through the M vs H loops at zero magnetization. The anisotropy field is only defined for hard axes, as it should be zero for the easy axes in theory. In practice, this is never the case, and therefore also values are determined for the easy axes of the thin films. Comparing the obtained values for the *H*_an_ for the phase pure LSMO film, 0.6 kA/m in the IP direction and 1106 kA/m in the OOP direction, confirms a clear easy axis in the IP direction as expected. For the (LSMO)_0.3_(ZnO)_0.7_ VAN film an *H*_an_ of 27 kA/m is determined for the IP direction, 2 orders of magnitude higher than for the pure LSMO film, while a value of 310 kA/m is determined for the OOP direction, about four times lower than the pure LSMO film. The difference between in-plane and out-of-plane is still large enough to conclude that the easy axis is oriented in-plane for VAN films with an LSMO contribution of 30%. As the LSMO contribution is further reduced to 10% in (LSMO)_0.1_(ZnO)_0.9_ VAN films, the vertical interfaces play an even larger role and *H*_an_ becomes 207 kA/m in the IP direction and 167 kA/m in the OOP direction. These values are of the same order of magnitude, and therefore, there is no clear easy and hard axis along the IP and OOP directions. However, since the OOP direction has a lower H_an_, the easy axis is slightly more aligned in the OOP than in the IP direction.

### 3.3 Shape- and Strain-Induced Magnetic Anisotropy

To elucidate the contributions of the shape- and strain-induced anisotropy to the magnetic response of the nanopillars in the VAN films, the theoretical contributions for the (LSMO)_0.1_(ZnO)_0.9_ thin film are determined and compared to the experimental results. The diameter of the LSMO nanopillars in the (LSMO)_0.1_(ZnO)_0.9_ VAN film is estimated to be about 15 nm. With a film thickness of about 265 nm, an aspect ratio of 18 is obtained, which corresponds to a negligible demagnetizing factor of *N*_*z*_ = 0.007.^33^ Since the sum of the demagnetizing factors in the *x*, *y*, and *z* directions must equal 1, this means *N*_*x*_ = *N*_*y*_ = (1 – *N*_*z*_)/2 = 0.5, where *x* and *y* lie in the plane of the thin film, perpendicular to the nanopillar axis. For magnetization in the OOP direction, the value of the shape anisotropy field can be calculated by
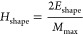
1where *H*_shape_ is the shape contribution to the anisotropy field in A/m, and *E*_shape_ is the associated energy density given by
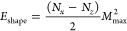
2where *M*_max_ is the maximum magnetization in the OOP direction in A/m. An anisotropy field of 359 kA/m is calculated for these nanopillars in the OOP direction, which is of the same order of magnitude as the experimentally obtained value of 167 ± 8 kA/m.

The strain-induced magnetic anisotropy field can be calculated through the following expression:
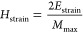
3where *E*_strain_ is the anisotropy energy associated with the stresses in the LSMO phase as a result of the strain and can be expressed as^34^
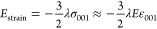
4where λ is the magnetostriction coefficient, *E* is Young’s modulus, and ε is the strain in the OOP direction. At the LSMO:ZnO vertical interface a maximum theoretical OOP tensile strain of 0.46% is present, assuming a 5:6 domain matching model^16^ with lattice parameters of 3.87 and 3.24 Å for LSMO and ZnO in the out-of-plane direction (19.35 and 19.44 Å), respectively. Assuming a fully strained LSMO phase, a magnetostriction coefficient^35^ of ∼25 × 10^–6^, and a Young’s modulus^36^ of 128 GPa, the associated *H*_an_ is calculated to be 4 kA/m. This contribution is negligible to the estimated *H*_an_ of 359 kA/m associated with the shape anisotropy. This result indicates that in LSMO:ZnO VAN films the main contribution to the magnetic anisotropy comes from the shape of the nanopillars and not from the strain induced in the LSMO phase. The difference between the experimentally obtained values for the anisotropy fields and the calculated values can be explained by the nonideal nature of the VAN films exhibiting variations in the nanopillar dimensions and ordering.

The dominating contribution of the shape to the magnetic anisotropy in these VAN films is in strong contrast to previous studies on LSMO nanowires exhibiting easy axes perpendicular to the nanowire axis due to substrate-induced strain effects.^14^ Additionally, in similar vertically aligned nanocomposite thin films of BaTiO_3_:CoFe_2_O_4_, it was shown that the OOP easy axis was mainly the result of strain effects, with only a small contribution of the shape.^37^ A significant difference is the lower aspect ratio of about 6 for the BaTiO_3_:CoFe_2_O_4_ VAN films, resulting in a lower shape anisotropy contribution, which in combination with the higher magnetostriction coefficient of CoFe_2_O_4_ resulted in a dominant strain-induced anisotropy contribution.

There are several approaches to further increase the magnetic anisotropy of such nanopillar-matrix architectures. The first option is to increase the aspect ratio since this will also increase the shape anisotropy field. However, since the current aspect ratio already leads to a negligible demagnetizing factor, any further increase is not expected to have a significant effect. The second option is to increase the strain-induced anisotropy by increasing the out-of-plane tensile strain at the vertical LSMO:ZnO interfaces. However, this seems not possible, as the lattice parameters of the involved materials already lead to a specific epitaxial matching. The final option is to increase the magnetostriction coefficient of LSMO, which has been achieved previously by 1 order of magnitude through doping with Tb on the La site.^38^ Although this would result in a higher anisotropy field of about 40 kA/m, this value is still 1 order of magnitude lower than the theoretical shape anisotropy field of 359 kA/m.

## 4 Conclusion

Vertically aligned nanocomposites composed of self-assembled ferromagnetic La_0.7_Sr_0.3_MnO_3_ nanopillars in a surrounding ZnO matrix are investigated for a controllable magnetic anisotropy. The nanopillar-matrix architecture of the VAN films, with the introduction of large ZnO regions within a LSMO thin film, breaks the typical shape anisotropy with an in-plane easy axis and makes the alignment of the magnetic domains (i.e., nanopillars) along the out-of-plane direction easier. Confinement of LSMO into nanopillar dimensions down to 15 nm in (LSMO)_0.1_(ZnO)_0.9_ VAN films aligns the magnetic easy axis along the out-of-plane (i.e., perpendicular) direction, in strong contrast to the typical in-plane easy axis for strained, phase pure LSMO thin films. The dominant contribution to the magnetic anisotropy in these (LSMO)_0.1_(ZnO)_0.9_ VAN films comes from the shape of the nanopillars, while the epitaxial strain at the vertical LSMO:ZnO interfaces exhibits a negligible effect. The large aspect ratio of about 18 for the LSMO nanopillars in such (LSMO)_0.1_(ZnO)_0.9_ VAN films leads to a negligible demagnetizing factor in the out-of-plane direction and, therefore, results in a strong shape anisotropy field almost 2 orders of magnitude larger than the strain anisotropy field.

The vertically aligned nanocomposite (LSMO)_0.1_(ZnO)_0.9_ films with their large remnant magnetization of 2.6 μB/Mn and a coercive field of 116 kA/m could be an interesting strategy for robust data storage applications when the LSMO nanopillars can be switched individually. The one-step self-assembly process involved in creating these VAN films would be much simpler than the multistep processes used nowadays to produce memory devices. The projected information density can be determined by assuming that one magnetic nanopillar can carry one bit of information. For cylindrical nanopillars with an average diameter of 15 nm, the average volume that one LSMO nanopillar and its surrounding ZnO matrix contain can be calculated using a volumetric ratio of 21.2:78.8 for LSMO:ZnO in (LSMO)_0.1_(ZnO)_0.9_ VAN films. This corresponds to an area of 834 nm^2^ per ferromagnetic nanopillar, which equals a maximum theoretical bit density of 0.12 Tbits/cm^2^ (i.e., 0.77 Tbits/inch^2^), very close to a recent hard drive density of 1 Tbits/inch^2^.^39^ Optimization of the nanopillar-matrix architecture as well as the involved compounds could lead to further improvement of the magnetic anisotropy toward enhanced data storage.
